# Identification of SNPs associated with muscle yield and quality traits using allelic-imbalance analyses of pooled RNA-Seq samples in rainbow trout

**DOI:** 10.1186/s12864-017-3992-z

**Published:** 2017-08-07

**Authors:** Rafet Al-Tobasei, Ali Ali, Timothy D. Leeds, Sixin Liu, Yniv Palti, Brett Kenney, Mohamed Salem

**Affiliations:** 1 Computational Science Program, Middle Tennessee State University, Murfreesboro, TN 37132 USA; 2 Department of Biology and Molecular Biosciences Program, Middle Tennessee State University, Murfreesboro, TN 37132 USA; 3 National Center for Cool and Cold Water Aquaculture, ARS-USDA, Kearneysville, WV 25430 USA; 4 Division of Animal and Nutritional Sciences, West Virginia University, Morgantown, WV 26506 USA

**Keywords:** Rainbow trout, Fish, SNPs, Genetic markers, RNA-Seq

## Abstract

**Background:**

Coding/functional SNPs change the biological function of a gene and, therefore, could serve as “large-effect” genetic markers. In this study, we used two bioinformatics pipelines, GATK and SAMtools, for discovering coding/functional SNPs with allelic-imbalances associated with total body weight, muscle yield, muscle fat content, shear force, and whiteness. Phenotypic data were collected for approximately 500 fish, representing 98 families (5 fish/family), from a growth-selected line, and the muscle transcriptome was sequenced from 22 families with divergent phenotypes (4 low- versus 4 high-ranked families per trait).

**Results:**

GATK detected 59,112 putative SNPs; of these SNPs, 4798 showed allelic imbalances (>2.0 as an amplification and <0.5 as loss of heterozygosity). SAMtools detected 87,066 putative SNPs; and of them, 4962 had allelic imbalances between the low- and high-ranked families. Only 1829 SNPs with allelic imbalances were common between the two datasets, indicating significant differences in algorithms. The two datasets contained 7930 non-redundant SNPs of which 4439 mapped to 1498 protein-coding genes (with 6.4% non-synonymous SNPs) and 684 mapped to 295 lncRNAs. Validation of a subset of 92 SNPs revealed 1) 86.7–93.8% success rate in calling polymorphic SNPs and 2) 95.4% consistent matching between DNA and cDNA genotypes indicating a high rate of identifying SNPs with allelic imbalances. In addition, 4.64% SNPs revealed random monoallelic expression. Genome distribution of the SNPs with allelic imbalances exhibited high density for all five traits in several chromosomes, especially chromosome 9, 20 and 28. Most of the SNP-harboring genes were assigned to important growth-related metabolic pathways.

**Conclusion:**

These results demonstrate utility of RNA-Seq in assessing phenotype-associated allelic imbalances in pooled RNA-Seq samples. The SNPs identified in this study were included in a new SNP-Chip design (available from Affymetrix) for genomic and genetic analyses in rainbow trout.

**Electronic supplementary material:**

The online version of this article (doi:10.1186/s12864-017-3992-z) contains supplementary material, which is available to authorized users.

## Background

Fish growth rate, muscle yield and fillet quality are major traits affecting profitability of aquatic food animal production. As feed cost is a major factor influencing the profitability, efficiency of growth is important and related to growth rate and muscle yield and composition. Skeletal muscle constitutes about 50–60% of the fish weight [1]. Given that growth efficiency and fillet firmness and appearance are critical for profitability and production of premium products [2], optimizing fish growth, muscle yield and fillet quality traits is a key objective in aquaculture breeding programs. Traditional phenotype-based selection is typically used to select for fast growth; however, muscle yield and quality traits are difficult to improve by conventional selection because measurement of these traits requires sacrificing the animal [2].

Genomic selection tools have been created to improve economically important traits in plants and livestock. Genetic maps, which characterize the linkage or co-inheritance patterns of genetic markers, have been developed for a wide range of species, including fish, with the aim of discovering allelic variation affecting traits; and ultimately identify DNA sequences underlying phenotypes [3, 4]. Markers have been identified by various molecular techniques, including numerous and genome-wide single nucleotide polymorphisms (SNPs). In addition, recent technological developments have enabled high throughput genotyping of these SNPs rendering them useful for genome-wide association studies [5–8]. Functional SNPs are generally defined as SNPs from genome sequences that affect structure, expression or function of a gene. These sequences include coding SNPs (e.g. non-synonymous, splicing), promoter and noncoding SNPs, as well as functional elements identified from studying of genome conservation [9]. Coding/Functional/ SNPs (c/fSNPs) are especially important because they have the potential to change the function of a protein [4, 10, 11]. In addition, c/fSNP markers, because they are located within expressed genes, they are unlikely to become unlinked from their associated genes due to genetic recombination. Therefore, c/fSNPs can be useful genetic markers for detecting significant associations with phenotypes. Understanding molecular mechanisms of muscle growth and quality can help in making better selection decisions. In terrestrial livestock, several genes, genetic markers and QTLs associated with production traits, including growth, have been characterized using molecular techniques [12, 13]. In addition, marker-assisted selection has been used to enhance genetic improvement in livestock breeding programs by direct selection on genes affecting economic traits [14] and to optimize selection for quantitative traits [12, 13]. However, the genetic basis of muscle growth and quality traits is not well studied in fish [15].

Rainbow trout is the most cultivated cool and cold freshwater fish in the U.S. [16], and it is considered a model species for studies in several fields of biology, including ecology [17], pathology [18], physiology [19], toxicology [20] and carcinogenesis [21]. Several studies used RNA sequencing to identify markers in human [22, 23] and non-model species [11, 24, 25]. However, most SNP detection algorithms were developed for DNA-Seq analyses and are not optimized/tested for RNA-Seq, especially in pooled samples. The objective of this study was using RNA-Seq analyses of pooled samples to identify c/fSNP markers and develop a resource for studies of marker association with production traits in rainbow trout. First, transcriptome-wide SNP allele frequencies were correlated to phenotypic variations in fish whole body weight (WBW) and muscle yield, fat content, shear force and whiteness. Second, SNPs with allelic imbalance scores (ratios between the allelic frequencies of the high-end families and that of the low-end families) were identified. Then, a subset of the putative SNPs was validated for allelic polymorphism and tested for trait association. Finally, genes harboring SNPs with allelic imbalances were annotated to obtain insight into the potential functional effects of the SNPs.

## Result and discussion

### Phenotypes

SNPs were identified in fish families with divergent phenotypes in WBW, muscle yield, fat content, shear force (texture) and whiteness of the fillet. These rainbow trout were from a growth-selected line developed by the NCCCWA breeding program [26]. Briefly, this line was created through artificial selection, starting in 2004, from 7 founder strains with documented diversity and domestication history. Over five generations, the population responded to selection by 9.8–12.7% increase in WBW per generation, and rate of inbreeding averaged 0.86% per generation [26]. In this study population, which was sampled after three generations of selection (hatch year of 2010), WBW was positively correlated with muscle yield and muscle fat content (*R* ^*2*^ = 0.56 and 0.50 respectively, data not shown). Our previous reports showed that fast growth may be genetically associated with improved muscle yield, paler fillets (affected by intramuscular fat content) and firmer texture [27]. The trait heritability estimates for muscle yield, muscle weight, WBW10, WBW13, carcass weight, fat percentage, shear force and fillet color were moderate to high (0.31–0.81) [6, 27]. Those moderate to high heritability estimates imply that substantial additive genetic variation exist in the study population for growth and carcass traits.

**Fig. 1:**
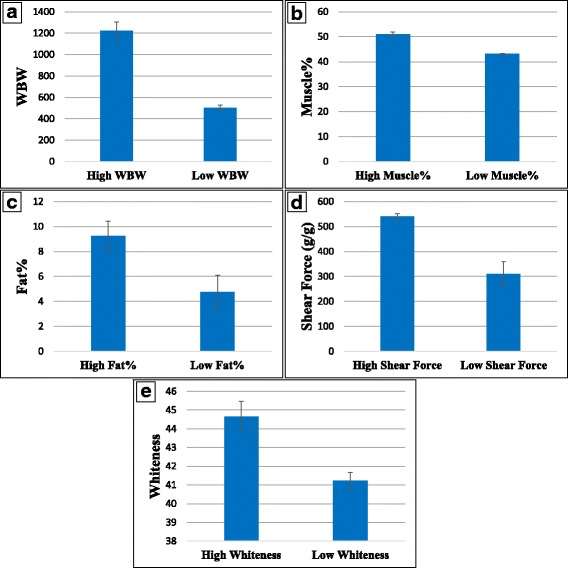
Phenotypic variations in fish families with contrasting phenotypes for five different traits; whole-body weight (**a**), muscle yield (**b**), fat content (**c**), shear force (**d**) and fillet whiteness index (**e**). All differences were statistically significant (*p* < 0.01)

For RNA sequencing, muscle samples were collected from 7 to 9 different full-sib families showing divergent phenotypes per trait (i.e. 3–5 high ranked families versus 3–5 low ranked families per trait). Five fish were sampled from each family. Divergent phenotypic attributes (Fig. 1) were statistically different (*P* < 0.01): WBW (1221.6 g ± 84.25 vs. 502.1 ± 28.0 g), muscle yield (50.9% ± 1.6 vs. 43.3% ± 2.3), muscle crude-fat (9.24% ± 1.2 vs. 4.77% ± 1.3), shear force (grams force/g of sample; 539.64 ± 12.3 vs. 310.01  ± 49.2), and fillet whiteness index (44.7 ± 0.8 vs. 41.23 ± 0.4) for high- vs. low-ranking groups, respectively. Means and standard deviations of these traits were calculated from the family averages.

### Identification of putative SNPs

RNA pools from muscle tissues of 5 fish per family were used for RNA-Seq analyses. A total of 259,634,620 reads (100 bp single-end) were generated from 22 families at an average of 11,801,573 reads per family. Reads were aligned against the rainbow trout genome [28] using the STAR [29] alignment tool. Percentage of reads mapped to the genome ranged from 80% to 82% per family.

A total of 204,604 putative SNPs were detected for the five traits using Haplotypecaller tool of Genome Analysis Toolkit v3.3.0 (GATK) [30], with an average of 40,920 SNPs per trait. Using the SAMtools/Popoolation software package [31, 32], a total of 304,805 putative SNPs were predicted, with an average of 60,961 SNPs per trait (Table 1). After removing redundant SNPs among all traits, we had 59,112 SNPs from GATK and 87,066 from SAMtools/Popoolation2 with 50,885 shared between the two bioinformatics pipelines (Table 1).

**Table 1:** Summary of putative SNPs and SNPs showing allelic imbalances identified by SAMtools and GATK for each trait

Trait	No. of putative SNPs		No. of SNPs with Allelic imbalance			
	SAMtools/Popoolation2	GATK	SAMtools/Popoolation2		GATK	
			0.5/2.0	0.0/1.0	0.5/2.0	0.0/1.0
Fat%	59,032	38,808	662	406	877	270
Shear	60,309	38,960	910	488	1152	261
Muscle%	61,117	42,383	1321	116	1507	76
Whiteness	64,636	44,460	1011	347	1283	298
WBW	59,711	39,993	1166	93	1456	64
Total # SNPs	304,805	204,604	5070	1450	6275	969
Total # SNPs non-redundant	87,066	59,112	4962		4798	
Total Common SNPs	50,885		1829			
	All putative SNPs(MAF > 0.05) =95,234*		Total No. of SNPs with allelic imbalance = 7930**			

Allelic imbalances were calculated at >2 for amplification and <0.5 for loss of heterozygosity. SNPs explicitly existing in only the high or low phenotypic group are indicated in the table by the 0.0/1.0 ratio. * 59 SNPs were multi-allelic, showing different alleles in association with different phenotypes. ** 1 SNP was multi-allelic showing different alleles predicted by different pipelines

After identifying putative SNPs, an in-house Perl script was used to estimate allelic imbalances of the SNPs in each trait. A total of 6275 SNPs with allelic imbalances were identified from the GATK dataset at cutoff values of >2.0 as an amplification and <0.5 as loss of heterozygosity. In addition, 969 SNPs explicitly existed in only the high or low phenotypic group. After removing redundant SNPs between traits at the two cutoff values, there were 4798 unique SNPs (Table 1). Similarly, SAMtools/Popoolation2 identified 5070 SNPs with allelic imbalances at cutoff values of >2.0 as an amplification and <0.5 as loss of heterozygosity. In addition, 1450 SNPs existed in families at one of the two ends of each trait variation scale but not in the other (Table 1). There were 4962 non-redundant SNPs among the five traits that were identified with SAMtools/Popoolation2 at the two cutoff values. There were only 1829 non-redundant SNPs shared between GATK and SAMtools/Popoolation2. Differences in variant calling and filtering steps might have caused the observed differences in number of SNPs between GATK and SAMtools/Popoolation2. There were 7930 non-redundant SNPs with allelic imbalances from both methods. The results of the SNPs’ allelic imbalances should be taken with caution because we could not find a reliable statistical test associated with the ratio calls derived from the allelic imbalance calculation to report statistical significance. However, by utilizing exact allele counts instead of frequencies, we were able to assign Chi Square *P*-Values to most of the SNPs with allelic imbalances. Out of the 7930 SNPs with allelic imbalances, there were 6038 SNPs with available read count for both alleles in the divergent families. Alternatively, there were 1892 with counts for only one allele. These SNPs existed in families at one of the two ends of each trait variation scale but not in the other. We performed chi-square test on the 6038 SNPs and found 5330 SNPs (83%) with P_value <0.05 and 710 SNPs (17%) with *P*_value greater >0.05 (Additional file 1).

For subsequent analyses, we combined SNPs from GATK and SAMtools/Popoolation2 into three different groups: 1) Non-redundant SNPs with allelic imbalances from both methods (7930 SNPs); 2) Common putative SNPs from both methods (50,885 SNPs); 3) Putative non-redundant SNPs from both methods (95,234 SNPs) (Table 1). All SNPs data are provided in Additional file 1.

### SNP validation

A total of 92 putative SNPs including 88 SNPs from the GATK/SAMtools common pool (50,885 SNPs) were selected for SNP validation. Among the 92 putative SNPs, 68 SNPs showed allelic imbalances (Table 2), including 25 SNPs identified by GATK pipeline, 10 SNPs identified by SAMtools pipeline, and 33 SNPs identified by both pipelines (Table 2). Among the 92 tested SNPs, 72 (78.2%) SNPs were polymorphic, 11(11.9%) SNPs were monomorphic and 9 failed the assay (Table 2). Failure of the Fluidigm assay can be caused by unsuccessful or non-specific primer binding to the target genomic DNA. Therefore, we cannot assume that a failed assay indicates failure of our bioinformatics pipeline to detect a SNP in the RNA sequence data, and can remove the failed SNP assays from the calculation of SNP validation rate. As 72 out of the 83 working Fluidigm SNP assays were polymorphic, we can claim 86.7% validation rate in detecting polymorphic SNPs in the overall putative SNP pool and 90% validation rate in the GATK/SAMtools shared SNPs pool. This success rate is much higher than what we previously achieved in rainbow trout using RNA-Seq (70%) and genomic reduced representation libraries (48%) [11, 33]. The improved success rate in this study is perhaps due to use of a reference genome instead of de novo assembled references used in the previous studies. In addition, a transcriptome sequence coverage of ∼7.4X per fish was used compared to only ∼0.97X in our previous RNA-Seq study [11]. The 90% successful SNP validation rate is comparable to that reported in diploid fish or using genomic RADs and doubled haploid fish in rainbow trout [7, 34]. In addition, a recent rainbow trout genome re-sequencing study with at least 10× genome coverage per fish had 86% successful validation rate [7]. Relatively lower success rates in SNP detection were reported from RNA-Seq studies in rainbow trout due to genome duplication and assembly errors in the genome/transcriptome references [11, 35, 36]. Noteworthy and in a separate study, we found variation in gene expression in only 75 genes distributed between all 5 traits (data will be published elsewhere). Therefore, differential gene expression effects on estimating allelic imbalances were negligible as only 75 genes distributed between all five traits were differentially expressed between the high and low families. Minor effects of variation in gene expression on allele frequency estimation accuracy were previously reported [37]. The SNP validation data, albeit small, indicated that the GATK method was more successful in calling polymorphic SNPs with allelic imbalances than the SAMtools pipeline; 87.5% versus 66.7%, respectively. However, combined GATK and SAMtools data had a 93.8% success rate. Success rates between SNPs with and without allelic imbalances were 88.7% and 86.7%, respectively. Importantly and out of 72 validated SNPs, 61 (84.7%) and 58 SNPs (80.5%) were polymorphic in fish from two different commercially important rainbow trout populations in the US, Troutlodge Inc. and Clear Springs Foods Inc., respectively. These results suggest that the SNPs identified in this study are also useful for other commercial rainbow trout populations.

**Table 2:** Number of putative and validated SNPs from each dataset

SNP Group	Total SNPs	Polymorphic	Monomorphic	Failed assay	Success rate
All putative SNPs (95,234)	92	72	11	9	86.7%
GATK/SAMTool common SNPs (50,289)	88	72	8	8	90.0%
Total SNPs with allelic imbalance	68	55	7	6	88.7%
GATK unique SNPs with allelic imbalance	25	21	3	1	87.5%
SAMTool unique SNPs with allelic imbalance	10	4	2	4	66.7%
GATK/SAMTool common SNPs with allelic imbalance	33	30	2	1	93.8%

To evaluate ability of the pipeline in calculating allelic imbalances, DNA and cDNA of the 35 fish used for RNA-Seq analyses of high versus low muscle yield were also genotyped. For all 72 validated SNPs, all DNA and cDNA genotypes were consistent except for 4.64% that indicated mono-allele specific gene expression as explained below.

### Assessment of mono-allelic gene expression

Out of the 72 validated polymorphic SNPs (Table 2), there were 46 SNPs that showed potential mono-allelic expression in cDNA in at least one fish. In other words, the genomic DNA is heterozygous for the SNP while cDNA is monomorphic. Thirty-three of the 35 fish showed mono-allelic expression in at least one SNP. Out of the aforementioned 46 SNPs, 5 SNPs were randomly selected for validation using Sanger sequencing. All SNPs were heterozygous at the DNA level. However, manual investigation of the cDNA sequence chromatograms exhibited existence of substantial allelic imbalances ranging from existence of two alleles with >2.0 X peak height ratios between the 2 alleles at the SNP base to a complete mono-allelic expression (a single peak). Overall, approximately 4.64% random mono-allelic/allelic imbalances existed in gene expression of rainbow trout. These data are consistent with a recent study in human stem cells showing that most allelic imbalances did not represent ‘on/off’ events, but instead revealed biased expression from each allele [38]. None of the 8 tested families in our study showed mono-allelic expression in all individuals specific to a given family, indicating no parental origin effect through genomic imprinting. Likewise, the human stem cell study suggested that most of the allele-biased gene expression is not due to genomic imprinting [38]. Compared to our estimated 4.64% mono-allelic expression, recent studies showed 12–24% random mono-allelic expression in mammals and 7–9% in interspecies catfish [4, 39–41]. Our mono-allelic expression assessment is based on only 72 SNPs, and hence a genome-wide assessment of mono-allelic expression in rainbow trout warrants further investigation.

### SNP genomic/functional classification

Three sets of SNPs were considered for genomic/functional classifications. For the 7930 SNPs with allelic imbalances, 2898 (37.69%) were intergenic. Of them, 635 (8.01%) and 721 (9.09%) SNPs were located within 5Kb upstream or downstream of protein-coding genes, respectively. The rest of the intergenic SNPs, 1633 (20.59%) were located more than 5Kb distant to protein-coding genes.

On the other hand, 4941 (62.31%) SNPs were genic, including 214 (2.70%) that were located within the 5′ untranslated region (5’UTR) and 1677 (21.15%) that were located in the 3′ untranslated region (3’UTR) of protein coding genes. In addition, 2548 (32.13%) SNPs were located within coding DNA sequences (CDS) and 502 (6.33%) SNPs were located within introns. Of the CDS SNPs, 504 (6.36%) were non-synonymous; 4 of these caused early stop codon, and 500 caused amino acid substitution (Table 3). There were 684 (8.63%) SNPs located within 295 lncRNAs (Table 3).

**Table 3:** Summary of SNPs classification for different SNP sets

Functional Class	SNPs with allelic imbalance 7.9 K	%	GATK/SAMtools Common SNPs 50.8 K	%	All putative SNPs 95.2 K	%
Intergenic	2989	37.69%	20,356	40.00%	46,901	49.25%
Intergenic(>5 K)	1633	20.59%	10,554	20.74%	27,651	29.03%
Upstream (<5 K)	635	8.01%	4594	9.03%	9005	9.46%
Downstream (<5 K)	721	9.09%	5208	10.23%	10,245	10.76%
Genic	4941	62.31%	30,529	60.00%	48,333	50.75%
5’UTR	214	2.70%	1389	2.73%	2247	2.36%
3’UTR	1677	21.15%	10,259	20.16%	16,420	17.24%
CDS	2548	32.13%	15,178	29.83%	22,616	23.75%
Intronic	502	6.33%	3703	7.28%	7050	7.40%
Non-synonymous	504	6.36%	3919	7.70%	5853	6.15%
Stop gain	4	0.05%	50	0.10%	79	0.08%
Missense	500	6.31%	3869	7.60%	5774	6.06%
LncRNA	684	8.63%	4386	8.62%	10,465	10.99%
Total number/percentage	7930	100.00%	50,885	100.00%	95,234	100.00%

Regarding the GATK/SAMtools shared SNPs (50,885 SNPs), there were 20,356 (40.00%) intergenic SNPs. Of these shared SNPs, 4594 (9.03%) were located within 5Kb upstream, and 5208 (10.23%) downstream of protein-coding genes. In addition, 10,554 (20.74%) were intergenic, more than 5Kb distant to protein-coding genes. In contrast, 30,529 (60.00%) SNPs were genic. And, 1389 (2.73%) of these SNPs were in the 5’UTR; 10,259 (20.16%) were in the 3’UTR, 15,178 (29.83%) were within CDS; and 3703 (7.28%) were within introns. Out of those within CDS SNPs, 3919 (7.70%) were non-synonymous SNPs. Fifty of these CDS SNPs were nonsense (causing premature stop codon), and 3869 (7.60%) were missense SNPs (Table 3).

Concerning all the putative SNPs, there were 46,901 (49.25%) intergenic SNPs. Of these, 9005 (9.46%) were located within 5Kb upstream; and 10,245 (10.76%) were downstream of protein-coding genes. In addition, 27,651 (29.03%) were more than 5Kb distant from protein-coding genes. Alternatively, 48,333 (50.75%) SNPs were genic, and of these genic SNPs, 2247 (2.36%) were in the 5’UTR; 16,420 (17.24%) were in the 3’UTR; 22,616 (23.75%) were within CDS; and 7050 (7.40%) were within introns. Of the CDS SNPs, 5853 (6.15%) were non-synonymous with 79 SNPs causing early stop codons and 5774 (6.06%) causing amino acid changes (Table 3).

In these three SNP datasets, there were large percentages of intergenic (including upstream/downstream) SNPs (37–49%). Approximately 10% intergenic in addition to 30% non-coding SNPs were reported in humans from RNA-Seq data [42]. Our high percentages of intergenic SNPs may be partially explained by the incomplete annotation of protein coding genes and exons in the current version of the rainbow trout reference genome sequence [28].

### Distribution and density of SNPs in the genome

**Fig. 2:**
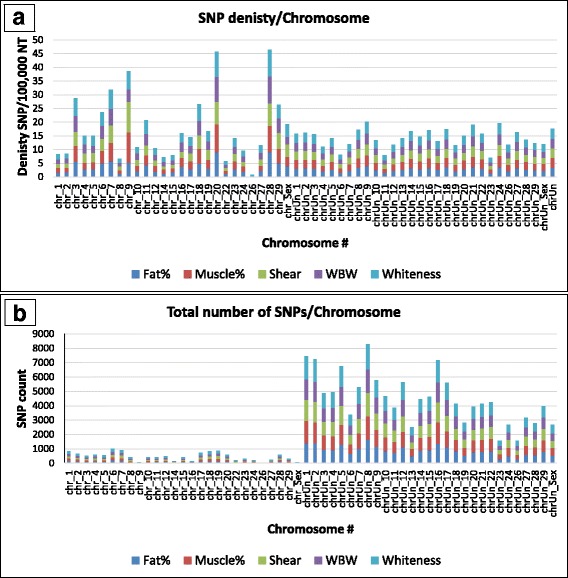
Genome distribution of the SNPs with allelic imbalances for all five traits. SNP density (SNPs per 100,000 NT) (**a**) and total number of SNPs (**b**) are shown for each chromosome. Chromosome “Unknown” (1.1 Gb scaffolds not assigned to chromosomes) had 4086 (49.05%) SNPs is not shown in the lower panel

Chromosome density distribution of the SNPs with allelic imbalances exhibited high density for all five traits in several chromosomes with the three highest peaks in chromosomes 9, 20 and 28 (Fig. 2a). All five traits revealed very similar pattern of distribution with a single exception; shear force exhibited a relative higher density than the other traits on chromosome 9. The similarity in density distribution between traits may be explained at least in part by the positive correlation that we observed between the phenotypes in this population. WBW and thermal growth coefficient were used as selection criterion in this population [11, 26], and we found that WBW as an independent variable has significant effects on muscle yield and fat percentage (multivariable regression analysis [*P* < 0.01], data not shown). However, despite the similarity in SNP density distributions, most of the identified SNPs were unique to each trait. From the 7930 SNPs with allelic imbalances, only 27 were shared by all five traits, 161 were shared by four traits, 680 were shared by three traits and 1783 were shared by two traits. In agreement with our results, a recent GWAS study identified two windows with effect on fillet yield located on chromosome 9 and explaining 1.0–1.5% of genetic variance in the same fish population [6].

As can be expected, the number of SNPs with allelic imbalances per chromosome was strongly correlated with chromosome length (Fig. 2b). In general, numbered unknown chromosomes, which are longer in the current reference genome [28], had more SNPs compared to the known chromosomes (Fig. 2b). Chromosome “Unknown” (1.1 Gb of scaffolds not assigned to chromosomes) had 4086 (49.05%) SNPs (not shown in Fig. 2b). Previous genetic mapping reports showed that the growth-related SNPs/QTL are distributed over ~20 chromosomes [11, 43, 44]. Together with our data, these reports confirm the polygenetic nature of growth/muscle related traits in rainbow trout.

### SNP functional annotation

**Fig. 3:**
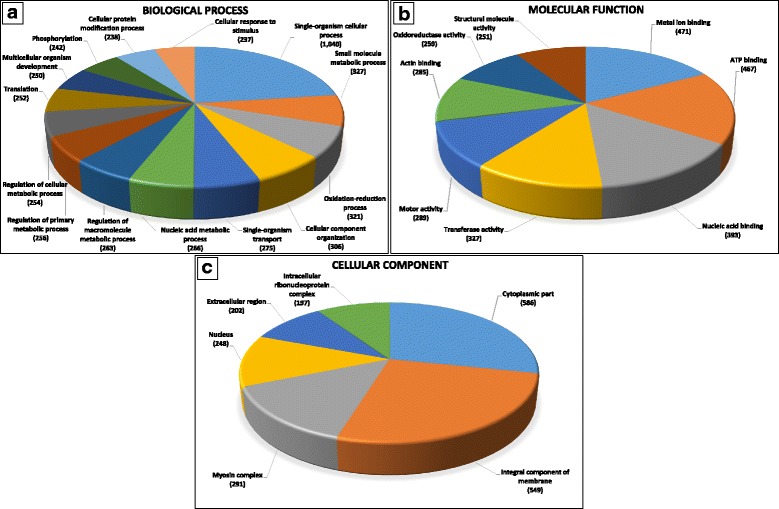
Gene Ontology (GO) assignment of the genes harboring SNPs with allelic imbalances in families with contrasting growth and muscle phenotypes. Genes were assigned GO terms according to their biological processes (**a**), molecular functions (**b**) and cellular components (c)

Functional annotation of genes harboring SNPs with allelic imbalances were performed using the Blast2GO suite [45]. The SNP-flanking sequences were searched against the NCBI nr-protein database using BLASTx; then, associated genes and Gene Ontology (GO) terms were acquired. In the biological processes category, SNP-harboring genes were associated with various cellular processes mainly involved in growth-related mechanisms, including regulation of metabolic and oxidation-reduction processes and protein translation (Fig. 3). In the molecular function category, SNP-containing genes were associated with binding metal ions, ATP, nucleic acid, and actin. In addition, a significant number of the genes were associated with transferase, motor, oxidoreductase, and structural molecule activities (Fig. 3). In the cellular component category, many of the genes exhibited association with the cytoplasmic compartment, membranes, myosin complex, and extracellular region compartment (Fig. 3). Genes with similar GO associated terms were previously reported to be involved in rainbow trout muscle growth and quality [11, 19, 43, 46–48].

Additionally, KEGG pathway mapping was used to assign enzyme function to the SNP-containing transcripts [49]. Searching transcripts against the KEGG database yielded 1043 transcripts (13.15%) with significant KEGG hits to 632 KEGG Orthologies (KOs) belonging to different pathways (Table 4). Most of the transcripts were assigned to growth-related metabolic pathways. There were 275 transcripts (182 KOs) related to metabolism. Under this category, sequences matching energy metabolism (88 transcripts, 57 KOs) appeared on the top of the list, with 52 transcripts (37 KOs) assigned to oxidative phosphorylation. Sequences matching carbohydrate metabolism occupied the second place (77 transcripts, 43 KOs) and were further classified into glycolysis/gluconeogenesis (39 transcripts, 18 KOs), citrate cycle (19 transcripts, 14 KOs) and pyruvate metabolism (16 transcripts, 10 enzymes). The next metabolic subcategories in the metabolic list were amino acid metabolism (56 transcripts, 41 KOs), lipid metabolism (27 transcripts, 22 KOs), and cofactors and vitamins metabolism (14 transcripts, 11 KOs). These preliminary SNP functional annotations are in agreement with previous reports that showed strong association between 1) mutations and altered expression of glycolytic and oxidative phosphorylation enzymes and 2) rainbow trout growth and muscle degeneration [11, 19, 43, 46, 47].

**Table 4:** KEGG biochemical mapping of the genes harboring SNPs with allelic imbalances in fish families showing contrasting growth and muscle phenotypes

	Total (all traits)		WBW		Muscle %		Fat %		Schear		Witness	
KEGG categories	No. of sequences (%)	No. of KOs	No. of sequences (%)	No. of KOs	No. of sequences (%)	No. of KOs	No. of sequences (%)	No. of KOs	No. of sequences (%)	No. of KOs	No. of sequences (%)	No. of KOs
Metabolism	275	182	133	99	130	96	102	77	125	92	142	105
Carbohydrate Metabolism	77 (28.00)	43	42(31.58)	28	39 (30.00)	27	26 (25.49)	19	39 (31.20)	25	47 (33.10)	28
*Glycollysis / Gluconeogenesis*	39	18	19	10	19	14	13	7	21	11	20	13
*Citrate cycle (TCA cycle)*	19	14	8	8	9	7	7	6	10	9	9	7
*Pyruvate metabolism*	16	10	6	4	7	7	5	4	10	8	6	5
*Pentose phosphate pathway*	13	5	8	5	7	5	7	3	3	2	3	3
Energy Metabolism	88 (32.00)	57	42 (31.58)	32	45 (34.62)	29	37 (36.27)	26	30 (24.00)	21	41 (28.87)	32
*Oxidative phosphorylation*	52	37	20	17	28	18	18	15	13	11	19	16
Amino Acid Metabolism	56 (20.36)	41	26 (19.55)	21	24 (18.46)	20	2 (20.59)	16	28 (22.40)	22	32 (22.54)	26
Lipid Metabolism	27 (9.82)	22	14 (10.53)	11	11 (8.46)	11	7 (6.86)	6	16 (12.80)	14	13 (9.15)	11
*Fatty acid degradation*	15	13	8	6	9	9	4	3	11	10	8	7
Metabolism Cofactors and Vitamins	14 (5.09)	11	4 (3.01)	4	6 (4.62)	4	5 (4.90)	5	5 (4.00)	5	4 (2.82)	4
Nucleotide Metabolism	13 (4.73)	8	5 (3.76)	3	5 (3.85)	5	6 (5.88)	5	7 (5.60)	5	5 (3.52)	4
Genetic Informatio Processing	176	112	69	50	79	59	50	40	83	59	74	55
Translation	105 (59.66)	69	36 (52.19)	31	48 (60.76)	39	30 (60.00)	27	45 (54.22)	35	47 (63.51)	40
*Ribosome*	68	48	25	23	32	26	21	19	33	27	32	28
*RNA transport*	22	12	9	6	11	9	7	6	8	4	7	6
Folding. Sorting and Degradation	62 (35.23)	38	28 (40.58)	17	24 (30.38)	16	19 (38.00)	12	30 (36.14)	19	23 (31.08)	13
*Protein processing in endoplasmic reticulum*	23	14	11	7	7	4	7	5	14	9	7	4
*RNA Degradation*	16	5	11	4	10	5	8	4	9	3	11	5
*Proteasome*	12	11	4	4	5	5	1	1	4	4	4	3
*Ubiquitin mediated proteolysi*	9	7	2	2	2	2	1	1	3	3	1	1
Transcription	9 (5.11)	5	5 (7.25)	2	7 (8.86)	4	1 (2.00)	1	8 (9.64)	5	4 (5.41)	2
*Spliceosome*	9	5	5	2	7	4	1	1	8	5	4	2
Environmental Information Processing	166	99	70	45	88	61	62	45	76	55	87	58
Signal Transduction	147 (88.55)	87	62 (88.57)	39	79 (89.77)	53	56 (90.32)	41	69 (90.79)	50	74 (85.06)	50
*P13K-Akt signaling pathway*	35	21	12	8	13	10	12	8	13	10	24	16
*Calcium signaling pathway*	36	18	16	9	13	8	14	10	16	12	16	11
*MAPK signaling pathway*	26	18	10	6	17	12	7	6	12	9	10	8
*cGMP-PKG signaling pathway*	26	16	10	7	8	8	7	6	10	8	12	10
*AMPK signaling pathway*	21	12	10	5	14	8	9	3	10	6	12	7
*cAMP signaling pathway*	18	12	6	5	4	4	7	6	8	7	7	6
*HIF-1*signaling pathway	11	9	4	2	7	4	3	3	9	6	8	4
*Hippo signaling pathway*	13	7	2	2	7	5	6	6	5	5	5	4
*FoxO signaling pathway*	7	6	3	3	2	2	3	3	1	1	3	3
*mTOR signaling pathway*	5	5	1	1	2	2	0	0	1	1	3	3
Signaling Molecules and Interaction	19 (11.45)	12	8 (11.43)	6	9 (10.23)	8	6 (9.68)	4	7 (9.21)	5	13 (14.94)	8
*ECM-receptor interaction*	17	10	7	5	7	6	6	4	7	5	13	8
*Cell adhesion molecules*	3	2	1	1	2	2	0	0	0	0	2	1
Cellular Processes	152	85	68	41	70	42	54	4	64	44	83	56
Cellular community	54 (35.53)	27	27 (39.71)	13	29 (41.43)	15	26 (48.15)	16	27 (42.19)	16	36 (43.37)	21
*Focal adhesion*	35	21	13	10	17	11	14	11	15	11	23	17
*Tight junction*	19	10	18	6	17	6	15	8	16	7	10	6
*Gap juction*	8	2	1	1	1	1	2	2	2	2	7	2
*Adherens junction*	5	3	4	3	5	3	3	3	5	3	3	3
Transport and Catabolism	42 (27.63)	24	17 (25.00)	11	20 (28.57)	14	8 (17.81)	8	16 (25.00)	11	18 (21.69)	11
Cell Growth and Death	36 (23.68)	22	16 (23.53)	11	1217.14	8	13 (24.07)	12	14 (21.88)	13	19 (22.89)	16
*Apoptosis*	19	13	13	7	5	4	6	6	7	7	11	9
*p53 signaling pathway*	7	5	6	4	3	1	1	1	3	3	4	4
Cell Motility	20 (13.16)	12	8 (11.76)	6	9 (12.86)	5	7 (12.96)	6	7 (10.94)	4	10 (12.05)	8
*Regulation of actin cytoskeleton*	20	12	8	6	9	5	7	6	7	4	10	8
Organismal Systems	274	154	108	66	124	84	109	81	122	82	129	89
Endocrine System	105 (38.32)	53	44 (40.74)	25	49 (39.52)	32	36 (33.03)	24	53 (43.44)	3	56 (43.41)	33
*Glucagon signaling pathway*	36	12	19	8	14	9	12	7	22	11	23	10
*Insulin signaling pathway*	32	12	14	7	11	6	7	4	12	6	22	10
*Thyroid hormone signaling pathway*	11	7	4	4	6	5	3	3	4	4	6	6
*Thyroid hormone synthesis*	6	4	3	2	1	1	3	3	2	2	1	1
Circulatory System	49 (17.88)	30	18 (16.67)	12	22 (17.74)	15	19 (17.43)	16	16 (13.11)	13	15 (11.63)	12
Immune System	44 (16.06)	28	16 (17.81)	10	21 (16.94)	14	22 (20.18)	17	24 (19.67)	16	23 (17.83)	17
Digestive System	32 (11.68)	16	13 (12.04)	9	9 (7.26)	7	18 (16.54)	11	13 (10.66)	9	20 (15.50)	14
*Protein digestion and absorption*	12	5	6	4	7	5	8	5	5	3	8	4
*Mineral absorption*	4	2	1	1	1	1	2	2	1	1	2	2
Nervous System	27 (9.85)	17	7 (6.48)	5	12 (9.68)	9	11 (10.09)	10	10 (8.20)	9	9 (6.98)	8
Aging	17 (6.20)	10	10 (9.26)	5	11 (8.87)	7	3 (2.75)	3	6 (4.92)	2	6 (4.65)	5
Total	1043	632	448	301	491	342	377	285	470	332	515	363

In addition, 176 KEGG annotated sequences were assigned to the genetic information processing category (112 KOs) that included translation (105 sequences, 69 KOs), folding, sorting and degradation (62 sequences, 38 KOs), and transcription (9 sequences, 5 KOs) (Table 4). A significant number of the SNP-harboring genes matched ribosomal (68 sequences, 48 KOs) and RNA-transport proteins (22 sequences, 12 KOs). Previously, we showed that the atrophying muscle and muscle from fast versus slow growing rainbow trout had differentially expressed genes involved in RNA processing, protein synthesis, posttranslational modification, and intracellular protein trafficking [19, 43, 46].

Moreover, 166 sequences (99 KOs) were classified by KEGG mapping into the environmental information processing category; these sequences were further assigned to signal transduction (147 sequences, 87 KOs) and signaling and interaction molecules (19 sequences, 12 KOs) (Table 4). The PI3K-Akt signaling, Calcium signaling, MAPK signaling, and cGMP-PKG signaling pathways had the largest numbers of hits: 21, 18, 18, and 16 KOs, respectively. Previous studies indicated involvement of MAPK and Calcium signaling in fish/muscle growth [46, 50].

Furthermore, the cellular processes category contained 152 KEGG-annotated sequences matching 85 KOs, which were further classified into cellular community (54 transcripts, 27 KOs), transport and catabolism (42 transcripts, 24 KOs), and cell growth and death (36 transcripts, 22 KOs) (Table 4). In the organismal systems category, the most significant subcategories were endocrine (105 transcripts, 53 KOs), circulatory (49 transcripts, 30 KOs), immune (44 transcripts, 28 KOs), and digestive systems (32 transcripts, 16 KOs). Recently, a GWAS study using the same fish population identified a small number of genes involved in muscle development explaining ~ 1.0% of the total genetic variance of the muscle yield and growth rate [6].

Distributions of KEGG matches were generally similar among all five traits. Albeit, we noticed an increased number of hits related to fillet whiteness compared to other traits, for carbohydrate metabolism (47 transcripts, 28 KOs) and amino acid metabolism (32 transcripts, 26 KOs) (Table 4). Similarly, there was a noticeable increase in numbers of hits in whiteness for PI3K-Akt signaling, focal adhesion, gap junction and regulation of actin cytoskeleton (Table 4). Regulation of focal adhesion and actin cytoskeleton were associated with development of pale, soft, and exudative (PSE) meat in turkey [51]. In addition, the muscle yield trait exhibited an increased number of transcripts for energy metabolism, with 28 transcripts/18 KOs belonging to oxidative phosphorylation. Shear force exhibited an increased number of transcripts belonging to lipid metabolism (16 transcripts, 14 KOs) (Table 4).

Our KEGG pathway mapping results have linked many of the genes harboring SNPs with allelic imbalances to potential regulation of growth and metabolic pathways, which may support pathway-based GWAS analyses in rainbow trout, similar to what has been recently applied to detect genetic pathways explaining live weight and muscle growth variation in cattle genotypes [52].

## Methods

### Fish population, sampling and sequencing

Phenotypic data and muscle samples were collected from ~500 fish representing 98 families (5 fish/family) from the growth-selected line at NCCCWA (year class 2010) as previously described [6, 11, 26]. Families were produced and reared until ~13 months post-hatch as described in reference [26]. Briefly, full-sib families were produced from single-sire × single-dam matings. Eggs were reared in spring water, and water temperatures were manipulated between approximately 7 and 13 °C to synchronize hatch times. Each family was stocked separately in 200-L tanks at a density of approximately 600 alevins/tank. Fish were randomly culled every month to maintain stocking densities <50 kg/m3. At about 5-months old, fish were anesthetized using 100 mg/L of tricaine methanesulfonate (Tricaine-S, Western Chemical, Ferndale, WA) and uniquely tagged by inserting a passive integrated transponder (Avid Identification Systems Inc., Norco, CA) into the dorsal musculature, and tagged fish were combined and reared in 1000-L communal tanks. Fish were fed a commercial fishmeal-based diet (42% protein, 16% fat; Ziegler Bros Inc., Gardners, PA) using automatic feeders (Arvotec, Huutokoski, Finland). Initially, young fish were fed at a daily feeding rate ∼ 2.5% of body weight (BW), which later was gradually reduced to approximately 0.75% of BW.

Fish were sampled as previously described for year class 2010 in Gonzalez-Pena et al., publication [6]. Briefly, WBW was measure in fish belonging to 98 families and families were sorted according to their WBW. The 2nd or 3rd fish from each family was selected for muscle sampling to keep the distribution of WBW consistently adjusted around the median of each family. Selected fish were randomly assigned to one of five harvest groups (~100 fish each) allowing one fish per family per harvest group. The five groups were sampled in five consecutive weeks (one group/week). Fish were samples at about ~13-months old (410–437 days post-hatch, mean body weight = 985 g; SD = 239 g). At harvest, fish were anesthetized in approximately 100 mg/L of tricaine methane sulfonate (Tricaine-S, Western Chemical, Ferndale, WA).

At harvest, a muscle sample was excised from the left dorsal musculature and frozen in liquid nitrogen for subsequent RNA sequencing. Fish were slaughtered, and eviscerated then head-on gutted carcasses were packed in ice, transported to the West Virginia University Meats Processing Laboratory (Morgantown, WV), and stored overnight. The next day, carcasses were hand-processed into trimmed, skinless fillets by a trained faculty member and weighed. Muscle yield and quality analyses were conducted as previously described [53]. Briefly, muscle yield was calculated as a percent of muscle weight relative to WBW. A 40 × 80 mm muscle section was separated, parallel to the long axis of the body, from the dorsal musculature for texture analysis [54]. The remaining muscle from the fillets was pulverized with liquid nitrogen in a Waring Blender (Waring, New Hartford, CT) and kept at −25 °C for chemical composition analyses. Proximate composition of muscle was determined using AOAC [55] approved methods. Crude fat was analyzed using the Soxhlet solvent extractor with petroleum ether. Texture of fillet sections was determined using a five-blade, Allo-Kramer shear cell attached to a Texture Analyzer (Model TA-HDi®; Texture Technologies Corp., Scarsdale, NY), equipped with a 50-kg load cell and at a crosshead speed of 127 mm/min. Force-deformation graphs were recorded and analyzed using the Texture Expert Exceed software (version 2.60; Stable Micro Systems Ltd., Surrey, U.K.). Peak shear force (g/g sample) was recorded.

Fresh fillet surface color was measured with a Chroma meter (Minolta, Model CR-300; Minolta Camera Co., Osaka, Japan) calibrated using a standard white plate No. 21333180 (CIE Y 93.1; × 0.3161; y 0.3326). L* (lightness), a* (redness), and b* (yellowness) values were recorded at three locations above the lateral line along the long axis of the right fillet, and these values were used to calculate a fillet whiteness index according to the following equation: Whiteness = 100 – [(100 – L)^2^ + a^2^ + b^2^]^1/2^ [81].

For RNA-Seq analyses, out of 98 families measured for phenotypic data, eight families (5 fish each) showing opposite phenotypes for each of the 5 traits were analyzed (4 high ranked families versus 4 low ranked families on average for each trait). Since some fish families were common between the traits, the total number of selected families for RNA-Seq was 22 families. Total RNA was isolated from each fish muscle sample using TRIzol™ (Invitrogen, Carlsbad, CA). Equal masses of total RNA from 5 samples of each family were pooled and used for RNA-Seq sequencing. cDNA libraries were prepared and sequenced on an Illumina HiSeq (single-end, 100 bp read length) using multiplexing standard protocols as previously described [56]. Briefly, mRNA was selected from one microgram of high quality total RNA. First-strand synthesis was synthesized with a random hexamer and SuperScript II (Life Technologies). Double stranded DNA was blunt-ended, 3′-end A-tailed and ligated to indexed adaptors. The adaptor-ligated double-stranded cDNA was amplified by PCR for 10 cycles with the Kapa HiFi polymerase (Kapa Biosystems, Woburn, MA) to reduce the likeliness of multiple identical reads due to preferential amplification. The final libraries were quantitated Qubit (Life Technologies, Grand Island, NY) and the average size was determined on an Agilent bioanalyzer DNA7500 DNA chip (Agilent Technologies, Wilmington, DE), diluted to 10 nM and the indexed libraries were pooled in equimolar concentration before sequencing.

### SNP detections using SAMtools/Popoolation2

For each trait (WBW, muscle yield, muscle fat content, shear force, and whiteness), sequence reads from each family were aligned to the rainbow trout genome using STAR [29]. After read alignment, the SAMtools view/sort and mpileup functions were used within the Popoolation2 package (version 1.201) to determine the genotype for each variant and calculate allele frequencies [57, 58]. Initial SNPs were considered at minimum reads >10 and minor allele count >4 and MAF > 0.05. Putative SNPs associated with each trait were determined by calculating SNP allelic imbalance scores as previously described [11, 59]. A SNP allelic imbalance score was determined by assessing the ratio of [frequency of allele A/frequency of allele B in high-end families]/[frequency of allele A/frequency of allele B in the corresponding low-end families]. The allelic imbalances score ranges from zero to infinity. SNPs with allelic imbalance were called if the ratio is more than or equal 2.0 (as an amplification) or less than or equal 0.5 (as loss of heterozygosity. The phase of the alleles could not be determined for families surveyed since the parental genotypes were not known for most of the fish. Allele counts in the divergent families were extracted from the VCF files. Chi-square test of two-by-two Tables [60] was performed with *p*-value <0.05 to determine if SNPs that are showing allelic imbalances are statistically significant.

### SNP detection using GATK tools

For the GATK pipeline [61], reads from each sample were aligned to the rainbow trout genome using STAR [29] as recommended by the GATK practice. Picard tools were used to sort the SAM files and to mark duplicates, a step used by GATK to reduce a false positive due to error in duplicate that could be falsely detected as a SNP. The following steps were performed according to GATK pipeline for RNA-Seq (Split and trim to reassign mapping quality, Indel realignment, local realignment around Indel in order to clean up any mapping artifacts and Base Quality Score Recalibration). After data preparation, variants were called using HaplotypeCaller followed by hard-filtering using the following parameters: Qual By Depth (QD) 2.0, FisherStrand (FS) 60.0: RMS Mapping Quality (MQ) 40.0, MAF > 0.05. Since GATK was not optimized to calculate allelic imbalances in RNA-Seq data, putative SNPs identified in each family were analyzed using an in-house Perl script to determine the allelic imbalances applying the criteria that we used in the SAMtools/Popoolation2 method.

### SNP validation

Flanking sequences (up to 250 bp on each side) of putative SNPs were extracted from the reference genome [28]. Some SNPs were removed from SNP assay design because either a sequence gap was located less than 60 bp from the SNP site or a non-target SNP was located less than 30 bp away from the target SNP. A total of 92 SNP assays were developed and evaluated with 282 DNA or cDNA samples. These included 85 DNA samples derived from 19 full-sib families used for RNA-Seq and their parents (38 DNA samples), DNA samples of 2 full-sib mapping families (2 parents and 19 offspring per family), 64 DNA samples from two commercial populations (Troutlodge Inc. and Clear Springs Foods Inc.) and 35 cDNA samples derived from the RNA samples used for RNA-Seq high versus low muscle yield. The SNP genotyping was performed following the instructions of the Fluidigm genotyping user guide. Briefly, DNA and cDNA samples were pre-amplified, diluted and used for genotyping with 96.96 Dynamic Array IFCs (Integrated Fluidic Circuits). The arrays were read using EP1 system, and genotypes were called automatically using Fluidigm SNP genotyping analysis software 4.1 with a confidence threshold of 85. The genotype clusters were examined for each assay and any wrong calls or no calls were corrected manually. The program Pedcheck [62] was used to identify genotypes inconsistent with Mendelian inheritance between parents and offspring. Chi-square goodness of fit tests were performed to identify SNPs with significant segregation distortion (*P* < 0.01) in the two mapping families. Those SNPs were reported as assay-failed SNPs.

For the Sanger sequencing validation of the SNPs showing potential mon-allelic gene expression, flanking sequences (up to 250 bp on each side) of each SNP were PCR amplified from DNAs and cDNA from the same 35 fish samples that were used for RNA-Seq high versus low muscle analyses. PCR amplicons were Sanger sequenced and manually inspected for consistency between DNA and cDNA genotypes or mono-allele specific gene expression as explained in the results section.

### Functional annotation of SNPs

SNP annotation by functional class (genic/intergenic etc.) for different SNP sets and their genome distributions were conducted using in-house Perl scripts. The gff file of the rainbow trout genome reference [28] was used to determine if a SNP is located within an mRNA start and end positions (genic), within a CDS, 5’UTR or 3’UTR. SNPs not within start and end positions of mRNA were considered intergenic. Upstream/ downstream intergenic SNPs were determined if located within 5 kb of an mRNA. SNPs within lncRNAs were determined using gtf file of our previously reported lncRNA reference [63]. Functional annotation of the SNP-harboring genes was performed using the Blast2GO suite [30] and KEGG pathway mapping.

## Additional file

Additional file 1: Putative SNPs and SNPs with allelic imbalances in association with total body weight, muscle yield, muscle fat content, shear force, and whiteness. SNP chromosome position, alleles, functional classification, associated gene ID and FASTA sequences are provided. (CSV 15332 kb)

## Abbreviations

CDS: Coding DNA sequences
FS: Fisher Strand
GATK: Genome Analysis Toolkit v3.3.0
GO: Gene Ontology
KOs: KEGG Orthologies
MAF: Minor allele frequency
MQ: Mapping Quality
PSE: Pale, soft, and exudative
QD: Qual By Depth
WBW: Whole body weight
